# Parasites alter food-web topology of a subarctic lake food web and its pelagic and benthic compartments

**DOI:** 10.1007/s00442-023-05503-w

**Published:** 2024-02-07

**Authors:** Shannon E. Moore, Anna Siwertsson, Kevin D. Lafferty, Armand M. Kuris, Miroslava Soldánová, Dana Morton, Raul Primicerio, Per-Arne Amundsen

**Affiliations:** 1 Department of Arctic and Marine Biology, Faculty of Biosciences, Fisheries and Economics, UiT The Arctic University of Norway, Tromsø, Norway; 2 U.S. Geological Survey, Western Ecological Research Center, at Marine Science Institute, University of California, Santa Barbara, CA USA; 3 Department of Ecology, Evolution, and Marine Biology, University of California, Santa Barbara, CA USA; 4 Institute of Parasitology, Biology Centre, Czech Academy of Sciences, Branišovská 31, 370 05 Ceske Budejovice, Czech Republic

**Keywords:** Parasite ecology, Food webs, Connectance, Trophic transmission, Trematoda

## Abstract

We compared three sets of highly resolved food webs with and without parasites for a subarctic lake system corresponding to its pelagic and benthic compartments and the whole-lake food web. Key topological food-web metrics were calculated for each set of compartments to explore the role parasites play in food-web topology in these highly contrasting webs. After controlling for effects from differences in web size, we observed similar responses to the addition of parasites in both the pelagic and benthic compartments demonstrated by increases in trophic levels, linkage density, connectance, generality, and vulnerability despite the contrasting composition of free-living and parasitic species between the two compartments. Similar effects on food-web topology can be expected with the inclusion of parasites, regardless of the physical characteristics and taxonomic community compositions of contrasting environments. Additionally, similar increases in key topological metrics were found in the whole-lake food web that combines the pelagic and benthic webs, effects that are comparable to parasite food-web analyses from other systems. These changes in topological metrics are a result of the unique properties of parasites as infectious agents and the links they participate in. Trematodes were key contributors to these results, as these parasites have distinct characteristics in aquatic systems that introduce new link types and increase the food web’s generality and vulnerability disproportionate to other parasites. Our analysis highlights the importance of incorporating parasites, especially trophically transmitted parasites, into food webs as they significantly alter key topological metrics and are thus essential for understanding an ecosystem’s structure and functioning.

**Supplementary Information:**

The online version contains supplementary material available at 10.1007/s00442-023-05503-w.

## Introduction

Food webs have been used for many decades in ecology to untangle the complicated relationships between predators and prey regulating ecosystem structure, function, and stability (Byers 2009; Thompson et al. 2012). However, the predator–prey relationship is only part of the story. Parasites are very common consumers in most systems and yet parasitism is often over-looked in food webs (Marcogliese and Cone 1997; Lafferty et al. 2008). There are numerous reasons to include parasitic interactions in a food web (Marcogliese and Cone 1997), and a growing field of work reflects this (Lafferty et al. 2006b; Hernandez and Sukhdeo 2008; Amundsen et al. 2009; Preston et al. 2014; McLaughlin 2018; Morton and Lafferty 2022). Parasites can have lasting impacts on their host organisms, both at the individual and population levels, by exploiting the host’s energy for its own development (Lafferty and Shaw 2013), altering the infected host’s behavior or morphology to increase its vulnerability to predation from free-living predators (Poulin and Thomas 1999; Miura et al. 2006), or driving trophic niche specialization and competitive release (or perhaps relaxation) (Hatcher et al. 2006; Britton and Andreou 2016; Rovenolt and Tate 2022). These specific parasitic characteristics can, therefore, also affect the rest of the ecosystem, with profound consequences for biodiversity and food-web complexity (Huxham et al. 1995; Lafferty et al. 2008; Amundsen et al. 2009; Thieltges et al. 2013; Banerji et al. 2015).

There are two main life cycle categories for parasites: the direct life cycle (monoxenous parasites) and the indirect or complex life cycle (heteroxenous parasites). Parasites employing a direct life cycle rely solely on a single definitive host species to complete their life cycle and reproduce (Dobson 1988). In contrast, parasites with a complex life cycle require at least one intermediate hosts species in addition to one definitive host species, and a parasite species may have more than one potential intermediate or definitive host species it can utilize (Anderson and Sukhdeo 2011; Baia et al. 2018). Hence, transmission from one host to the next is often dependent on trophic interactions in parasites with complex life cycles, thus indicating their importance when considering how food webs map trophic relationships (Anderson and Sukhdeo 2011; Baia et al. 2018). A key example of parasites with a complex life cycle is the trematodes, or flukes, that are parasitic flatworms with both sexual reproduction in vertebrate definitive hosts and asexual reproduction in mollusk intermediate hosts. These parasites are endoparasites that are found within the host as opposed to ectoparasites, which reside on the outside of the host (Baia et al. 2018). Trematode eggs are passed through the feces of its definitive host and hatch into miracidia, a free-living larval stage, that are hardwired to find the parasite’s first intermediate host. Once it penetrates the first intermediate host, the miracidium further develops and multiplies through asexual reproduction into cercariae, another free-living larval stage of the trematode parasite (Sukhdeo 2012). It is within this first intermediate host that the trematode larvae may usurp energy that the host has allocated to reproduction, essentially castrating the host (Lafferty and Kuris 2009b). Parasitic castration reduces the impact the parasite has on host viability while still accessing large amounts of energy for its own growth. Parasitic castration has also been reported in three-spined stickleback (*Gasterosteus aculeatus*, hereafter stickleback) by the tapeworm *Schistocephalus solidus* (Heins and Baker 2008; Barber and Scharsack 2009).

Trematode cercariae are released from their first intermediate hosts and use their brief (24–72 h) lifespan to seek out a suitable second intermediate host (Orlofske et al. 2015; McKee et al. 2020). While trematodes are specialists in respect to their first intermediate host, typically parasitizing mollusks, they are not host-specific in respect to their second intermediate and definitive hosts (Lafferty et al. 2006b; Preston et al. 2014). Finally, this second intermediate host is preyed upon by the parasite’s definitive host, allowing the trematode to infect this host and complete its life cycle. Such a complex life cycle allows for the introduction of numerous new interactions within a food web besides the classic parasite-host interactions. For example, within the intermediate host, trematodes prey upon other trematodes infecting the same host (Kuris 1990; Kuris and Lafferty 1994; Soldánová et al. 2012), free-living cercariae are susceptible to consumption by free-living predators (Thieltges et al. 2008; Johnson et al. 2010; Welsh et al. 2014 and all references therein, Born-Torrijos et al. 2020, 2021), and trematodes may also be vulnerable to concomitant predation during which the host they parasitize is consumed by another predator (Thieltges et al. 2013). Additionally, trematodes rely on trophic transmission between their second intermediate and definitive hosts, and these predator–prey interactions may be influenced by the parasite’s ability to alter the host’s behavior or morphology (Poulin and Thomas 1999; Miura et al. 2006). While this example includes both directly transmitted (via contact) and trophically-transmitted parasitism, it is clear that a parasite’s full life cycle should be represented in a food web to accurately describe their role in trophic interactions within a system.

The inclusion of parasites has been found to alter food-web structure and common food-web metrics, such as the number of links and trophic levels, food chain length, connectedness, and nestedness—factors that are all considered important for food-web complexity and stability (Lafferty et al. 2006b, 2008; Hernandez and Sukhdeo 2008; Amundsen et al. 2009; Thieltges et al. 2013; Morton and Lafferty 2022). As increasing numbers of studies have included parasites in community ecology studies, it is apparent that parasites’ wide diversity and unique functional roles alter our understanding of an ecosystem’s structure and functioning (e.g., Lafferty et al. 2008; Frainer et al. 2018; Morton and Lafferty 2022). Accordingly, excluding parasites and their links from food-web analyses may impede further developments of comprehensive food-web theory and understanding and may lead to erroneous conclusions concerning how ecosystems are structured and function.

Worldwide, only a few lacustrine systems have been subject to studies considering how parasites impact food-web ecology (Huxham et al. 1995; Amundsen et al. 2009, 2013; Preston et al. 2014), and none examined the role of parasites in the contrasting benthic and pelagic compartments within those systems. Benthic and pelagic habitats in lakes are likely to host different parasite communities that reflect each habitat’s physical properties, the species diversity and life history strategies of the compartments’ free-living communities, and the function of each compartment in the whole-lake ecosystem (Campbell et al. 1980; Marcogliese 2002). The open water characteristic of the pelagic habitat hosts a very particular and adapted set of free-living taxa consisting of phytoplankton, rotifers, crustacean zooplankton, fish, and birds. Meanwhile, the benthic habitat is populated by taxa using the more physically complex structures of the lakebed such as periphyton, macrophytes, insect larvae, benthic crustaceans, mollusks, worms, fish, and birds. Thus, parasitism and its effects may also vary across habitats. For instance, trematodes that use mollusks as first intermediate hosts are likely to be found in benthic food webs, whereas chytrid fungal parasites of phytoplankton are likely to be found in pelagic food webs. Furthermore, parasites might appear to affect food-web topology differently when habitats are combined rather than viewed in isolation. For instance, due to spatial separation, parasites in the combined food web should parasitize a smaller proportion of hosts than when considering each compartment separately.

To contrast parasites in the pelagic and benthic food webs, we added to existing studies about Takvatn, a subarctic lake in Norway. Studying the pelagic food web, Amundsen et al. (2009) addressed how parasites affected food-web structure and complexity, and Amundsen et al. (2013) examined how two fish introductions affected pelagic food-web topology and increased parasite diversity. Since those studies, additional work in the system has shown that the benthic compartment has a distinct set of free-living and parasitic taxa from the pelagic web (e.g., Kuhn et al. 2015; Frainer et al. 2016; Soldánová et al. 2017; Shaw et al. 2020; Prati et al. 2021). Here, we contrast key food-web metrics in each of Takvatn’s distinct compartments as well as the whole Takvatn system to understand how parasites affect food-web topology in a subarctic lake and to compare the pelagic and benthic food webs’ topology when including parasites. Specifically, we calculate the number of trophic levels, longest chain, linkage density, connectance, adjusted connectance, degree, generality, and vulnerability.

We hypothesize that the inclusion of parasites in the two compartments as well as the whole-lake food web would increase the number of trophic levels, chain length, linkage density, connectance and adjusted connectance, as seen in many other analyses of food webs with parasites across various types of systems (Table 1; e.g., Lafferty et al. 2006b; Hernandez and Sukhdeo 2008; Preston et al. 2014; McLaughlin 2018). Connectance is predicted to be lowest in the larger whole-lake web and highest in the smaller pelagic web because food-web connectance typically decreases with increasing food-web size (Riede et al. 2010). We predict adjusted connectance will also follow this trend as this metric more accurately measures the percentage of possible links that are observed (Lafferty et al. 2006a). Food-web generality and vulnerability are also predicted to increase along with free-living taxa generality and vulnerability as parasites represent additional consumers that rely on energy obtained from free-living taxa to continue their life cycles and introduce infective agents to top predators (Lafferty et al. 2008; Amundsen et al. 2009; Morton and Lafferty 2022). However, because each compartment has distinct parasitic communities due to their dissimilar free-living communities, we hypothesize that the generality and vulnerability of each compartment may behave differently from the other. We expect a larger increase in generality and vulnerability in the benthic compartment as we anticipate more general parasites with complex life cycles and broad host ranges, such as the speciose trematode community (Soldánová et al. 2017), to be prevalent here. Benthic trematodes also have a free-living life cycle stage during which they are extremely vulnerable to predation by free-living predators (Johnson et al. 2010; Born-Torrijos et al. 2020, 2021), and these trophic links also serve to increase benthic parasite vulnerability (Dunne et al. 2013; Morton and Lafferty 2022). The parasite community of the pelagic food web is in contrast dominated by cestodes (Amundsen et al. 2009), which expectedly are less speciose and not as general and vulnerable as the trematodes. As parasites are key species for ecosystem functioning and tightly woven within food webs (Lafferty et al. 2008; Frainer et al. 2018), it is vital to discern the impact parasites may have on trophic interactions and ecosystem structure, functioning, and stability within the key parts that make up the total ecosystem.

**Table 1:** Summary of the eight key topological metrics measured in three food-web comparisons of Takvatn’s pelagic and benthic compartments and the whole-lake web

Metric	Definition	Predicted direction	Prediction consistent with	Observed direction
Nodes	Taxa richness (*T*)	**↑**		**↑**
Observed links	Number of observed interactions between taxa in the web (*L*)	**↑**		**↑**
Trophic levels	Number of steps energy must take to reach a specific taxa from its source *TL*_*predator*_ *= 1 + ∑(TL*_*prey*_ *∗ 1*/number *of prey)*	**↑**	Lafferty et al. (2006a), Amundsen et al. (2009), Dunne et al. (2013), Preston et al. (2014), McLaughlin (2018), Morton and Lafferty (2022)	**↑**
Longest chain	Largest number of links between consumer taxa and basal taxa	**↑**	Lafferty et al. (2006a), Amundsen et al. (2009), Dunne et al. (2013), McLaughlin (2018), Morton and Lafferty (2022)	**↑ ↓**
Linkage density	Mean number of links per taxa *D* = *L*/*T*	**↑**	Lafferty et al. (2006a), Lafferty et al. (2006b), Hernandez and Sukhdeo (2008), Amundsen et al. (2009), Dunne et al. (2013), Preston et al. (2014), McLaughlin (2018), Morton and Lafferty (2022)	**↑**
Connectance	Porportion of potential interactions that are realized *C* = *L*/*L*_*p*_	**↑**	Lafferty et al. (2006a), Lafferty et al. (2006b), Hernandez and Sukhdeo (2008), Amundsen et al. (2009), Dunne et al. (2013), Preston et al. (2014), McLaughlin (2018)	**↑**
Adjusted connectance	Denominator for predator–prey + parasite-host subweb: number of free-living taxa * (number of free-living taxa + number of parasite taxa). Denominator for predator–prey + parasite-host + predation on free-living parasites: (number of all taxa)^2^—(number of parasite taxa)^2^* C =* *L/L*_*p.adj*_	**↑**	Lafferty et al. (2006a), Morton and Lafferty (2022)	**↑**
Mean degree	Average number of links (to prey/host taxa + predator/parasite taxa)	**↑**	Amundsen et al. (2009), McLaughlin (2018)	**↑**
Mean generality	Average number of out links (to prey/host taxa). Resources per consumer	**↑**	Marcogliese (1996), Køie (1993), Palm and Caira (2008)	**↑ = **
Mean vulnerability	Average number of in links (to predator/parasite taxa). Consumers per resource	**↑**	Lafferty et al. (2006a), Amundsen et al. (2009), Dunne et al. (2013), McLaughlin (2018), Morton and Lafferty (2022)	**↑ = **

↑ Represents an increase, ↓ represents a decrease, and ≠ represents no change. The observed direction symbols in black indicate where the observed results agreed with our predictions, while those in red indicate where the observed results did not agree with our predictions. Some observed directions include more than one symbol to reflect differences in the direction of change among the metrics of different compartments

## Materials and methods

### Study system

The Norwegian lake Takvatn (-vatn is the Norwegian word for lake) is a subarctic, oligotrophic, and dimictic lake that is located 300 km north of the Arctic Circle at 69°07’ N, 19°0 E. The lake itself is situated 214 m above sea level and has an area of 15 km^2^. Takvatn contains two main basins, each measuring over 80 m in depth (88 m at its deepest point). The littoral zone covers about 30% of the lake area and is generally characterized by a gentle slope from the upper to the lower littoral. The upper three meters are exposed, hard bottom substrate without microvegetation, followed by a vegetation belt dominated by *Nitella* (Chlorophyta) at about 3–12 m depth. Deeper parts of the lake are in the aphotic zone and characterized by sand and silt substratum. From late November to mid-January Takvatn experiences 24 h of darkness and from late May to late July, 24 h of daylight. The lake is typically ice-covered from November or December to May or June. The average air temperature is − 10 °C in January and 13.2 °C in July. The maximum epilimnetic water temperature is c. 14 °C (Prati et al. 2021). Surrounding Takvatn is a landscape of mountains, birch-dominant forest scattered with pine trees, and patches of farmland.

### Data collection and food-web matrix construction

The Takvatn food web is comprised of an *n* × *n* matrix consisting of n nodes (species life stages in this case) in which the columns represent consumers (predators and parasites) and the rows represent resources (prey and hosts) (see Cohen 1978; Lafferty et al. 2006b; Amundsen et al. 2009). Interactions between nodes are represented as links (either present or absent), and all links arise from detritus, phytoplankton, periphyton, and aquatic plants as the basal energy sources. Both free-living species and trophically- and non-trophically transmitted parasites are included in the food web. Free-living taxa are understood to be non-parasitic organisms involved in classic predator–prey interactions within a food web. The food web is spatially restricted to the lacustrine habitat, however, terrestrial inputs to the lake that are directly consumed by lake-dwellers and terrestrial predators that directly feed on lake organisms (i.e., eleven bird taxa and the sole mammalian species—*Neovison vison*, the mink) were included. Major sources of terrestrial input to the lake include terrestrial organic matter and terrestrial surface insects, which come directly from the terrestrial system and do not feed on anything in the freshwater system but are important in the diet of salmonid fish in the summer (Milardi et al. 2016; Prati et al. 2021). Furthermore, the food web is temporally restricted to include taxa and interactions occurring during the ice-free season of Takvatn.

### Species list

Takvatn has been extensively studied through a long-term ecological research program with annual surveys since 1980 (Amundsen et al. 2019). The current food web is an extension of a previously published food web for the pelagic compartment in Takvatn, which integrated information on pelagic species and feeding interactions in the lake from 1986 to 2007 (Amundsen et al. 2009). The benthic web was assembled using similar methods to the pelagic web and includes sampling from 1985 up to 2015. Additionally, ongoing data collection of the pelagic system allowed us to update the previously published pelagic web based on new findings. Most importantly, the large cladoceran *Bythotrephes longimanus* has emerged as an important food item for fish in the lake as a long-term effect of a culling of the Arctic charr (*Salvelinus alpinus*, hereafter charr) population (Skoglund et al. 2013; Prati et al. 2021) and was added to the previously published web. In the current food web, we have only included birds that have been observed at the lake over the past two decades (Klemetsen and Knudsen 2013). Thus, the long-tailed duck (*Clangula hyemalis*) and the red-throated diver (*Gavia stellata*) were excluded from the previously published food web, while the velvet scooter (*Melanitta fusca*) was added.

Direct observations were used to assemble the species list for both the previously published pelagic web and the benthic web added here (see Amundsen et al. 2009 for details), with some key differences aimed at capturing the different life histories of organisms in the benthic compartment. Both food webs were originally constructed using ontogenetic stages of species as nodes to account for trophic interactions occurring at different stages of a species’ life cycle. Most producers, consumers, and parasites were identified to the species or sometimes genus level. However, several groups of algae (periphyton and phytoplankton), bryophytes, terrestrial vegetation, terrestrial surface insects, nematodes, ostracods, and water mites (Hydracarina) are not resolved to the species or genus level because these groups have been less intensely studied or have a complex taxonomy. Similarly, most microorganisms are not included in the matrix, however, the most common species of algae, pelagic rotifers, and parasitic fungi were included due to their presumed importance (Kagami et al. 2007; Nowosad 2007; Guo et al. 2016). The species list was based on data from 40 years of annual sampling conducted at Takvatn and constructed using various sampling methods targeting specific taxa. Information on benthic species in Takvatn were compiled from several surveys in both the littoral and profundal benthic habitats of the lake, mainly targeting the southern basin. Macrophytes have been described in several publications (e.g., Klemetsen and Knudsen 2013; Frainer et al. 2016), while periphyton were identified exclusively for this study in 2013. Benthic invertebrates have been sampled in different types of habitats in the lake, and we included information from both soft and hard bottom in the littoral, as well as from the deep profundal habitat. Information on the composition of soft bottom invertebrates from the littoral to the profundal is mainly from the summer season in 2012 (Frainer et al. 2016). Hard-bottom invertebrates were sampled and identified in a 3-year survey in 2000–2003 (Klemetsen and Elliott 2010). More detailed taxonomic information on littoral cladocerans was included based on sampling from 1994 (Klemetsen et al. 2020). Some additional species were identified during sampling for the current study in 2012–2015. A variety of sampling methods is necessary for sampling benthic invertebrates in different substrates, and these are described in the aforementioned papers. For each sampling method, only free-living taxa that comprised > 1% of the total number of individuals sampled in a single year were included in the species list. However, there were a few taxa that failed the abundance criteria and yet were included in the species list due to their importance as food resources (several cladocerans and chironomids), hosts for parasites (two coleopterans, an ephemeropteran, and a plecopteran), or high-level predators (mink and some bird taxa).

An initial screening of parasite species present in the benthic web was obtained from continued annual parasite sampling through the long-term studies of the fish populations with occasional observations of parasites present in other organisms up to 2010 (Amundsen et al. 1997; Knudsen et al. 2001; Klemetsen and Knudsen 2013; Thieltges et al. 2013; Kuhn et al. 2016b; Henriksen et al. 2019; Prati et al. 2020b). However, the majority of information on parasites in the lake originates from 2012 to 2015 (with some additional sampling up to 2018), when the benthic invertebrate community, including arthropods, annelids, and mollusks, was more extensively sampled and screened for parasites (see Soldánová et al. 2017; Shaw et al. 2020). Helminths and other macroparasites have been thoroughly explored in our parasite surveys. While protozoans, fungi, bacteria, and viruses likely parasitize most species in our web, these groups are poorly studied, especially for invertebrates and aquatic producers. We only included some apicomplexan protozoan parasites on benthic invertebrates, common fungal infections in algae and copepods, and important ciliate (*Trichodina* sp.) and oomycete (*Saprolegnia* spp.) infectious in fish. All species identified in the parasite screenings were included in the Takvatn food web.

### Link assignment

The main trophic relationships included in this analysis were predator–prey, predator–parasite, parasite–host, and parasite–parasite. These four main categories of interactions were further split into twelve more specific link types (Table 2).

**Table 2:** Summary of the four link categories (consumer-resource) and the 12 link types observed in the pelagic and benthic compartments of the Takvatn food web and the whole-lake food web

Link category (consumer-resource)	Link type	Link description
Predator–prey	Predation	Consumer kills and feeds on more than one individual of the prey (resource) species
	Detritivory	Consumer feeds on or breaks down dead and decaying animal and plant matter
	Cannibalism	Special case of predation in which the consumer and resource are the same species
Predator-parasite	Predation on free-living, non-feeding stages	Consumer feeds on free-living parasite, but the parasite is not able to infect the consumer and is digested
	Concomitant predation	Consumer feeds on parasite living inside a prey, but the parasite is not able to infect the consumer and is digested
	Infection by predation on free-living, non-feeding stages	Consumer feeds on free-living parasite, and the parasite is able to infect the consumer
Parasite-host	Macroparasitism	Consumer (parasite) infects a host but does not necessarily cause the death of its host. The consumer (parasite) is not trophically-transmittable to other hosts
	Trophically-transmitted parasitism	Consumer (parasite) infects a host but does not cause the death of its host. The consumer (parasite) requires its host to be consumed by an appropriate predator host to complete its life cycle
	Pathogen infection	Consumer (parasite) infects a single host and multiplies within that host, often resulting in the death of the host
	Parasitic castration	Consumer (parasite) blocks the reproduction of the host
	Trophically-transmitted parasitic castration	Consumer (parasite) blocks the reproduction of the host and requires its host to be consumed by an appropriate predator host to complete its life cycle
Parasite-parasite	Parasite intraguild trophic interaction	Infection agent (parasite) attacks and kills (and often consumes) another infectious agent (parasite) within the same host

Predator–prey interactions between free-living taxa were mainly inferred from literature (81% of 2631 links between ontogenetic stages of species), with 48% of these links based on knowledge about the same species and 52% based on knowledge about similar species (typically within the same genus). These literature-based links consist mostly of pelagic and benthic invertebrates and birds. Key references included literature from other alpine or sub-arctic lakes in Norway and Scandinavia (e.g., Brittain 1978a, b; Larsson 1978; Lillehammer 1978a, b), in addition to more general literature for some groups (e.g., Monakov 1972; Nilsson 1997; Thorp and Covich 2009; Beaman and Madge 2010). Direct observations of feeding interactions in Takvatn were mainly discerned for the three fish species of charr, brown trout (*Salmo trutta*, hereafter trout), and stickleback, from which stomach contents have been analyzed (Amundsen and Klemetsen 1988; Klemetsen et al. 2002; Amundsen et al. 2007; Prati et al. 2020a, b, 2021; Sánchez-Hernández et al. 2022). We included the most important prey items, with a general rule to include prey with > 1% of volume and > 1% of frequency of occurrence in at least one month, from 1999 to 2018 for trout and charr and 2010–2016 for stickleback for different ontogenetic stages (three stages for trout and stickleback and five stages for charr). Additionally, more detailed information about charr and stickleback feeding on specific species of chironomids and benthic crustaceans was retrieved from samples from 1985 to 1988 (Klemetsen et al. 1992, 2003; Jørgensen and Klemetsen 1995). Prey information from both published studies and stomach content samples were sometimes provided at a coarser taxonomic resolution than the nodes used in this food web. In these cases, links with taxa in these wide groups were included only if the prey taxon was of “suitable” prey size and its habitat overlapped that of the predator.

Large field campaigns aiming to describe parasite species present in Takvatn have provided direct observations of many parasite-host links (46% out of a total of 780 links between stage-level nodes). These were mainly related to parasite species in fish, copepods, amphipods, and mollusks, for each of which most links were from observations (ranging from 55% in fish to 80% in Bivalvia). For benthic insect groups (i.e., chironomids, plecopterans, and coleopterans), 17% of all parasitic links were directly observed, while the majority (83%) were based on the insect species being suitable intermediate hosts for parasite species identified in the lake. For all identified parasites in Takvatn, literature reviews determined any additional hosts necessary to complete their life cycles, and these links were added to the food web (54% of all parasite-host links). Birds were not possible to sample and dissect in the study area, yet birds are known to be the final host for many parasite species present in Takvatn (e.g., several cestode and trematode species), therefore these links are the main source of the literature-based, parasite-host links (54% of all such links). The main literature source used for birds as final hosts was the Natural History Museum host-parasite database (https://www.nhm.ac.uk/research-curation/scientific-resources/taxonomy-systematics/host-parasites/database/index.jsp). 

Predator-parasite links represent free-living predators feeding on parasites. Free-living predators may feed deliberately on free-living parasites or accidentally on parasites within their prey (concomitant predation). Concomitant predation occurs when an infected prey item is eaten by a predator, but the parasite is not able to establish in the predator and so is merely digested and dies (Thieltges et al. 2013). All concomitant links in the current food web were inferred based on prey selection for predators (44% out of 2652 links for free-living predators feeding on parasites). Predation on free-living stages of parasites was inferred from the literature, which mainly focused on trematode cercariae (Thieltges et al. 2008; Johnson et al. 2010; Welsh et al. 2014 and all references therein), or chytrid zoospores (Kagami et al 2004, 2007; Rasconi et al 2011). We also considered identified predator–parasite links in previously published food webs (Thieltges et al. 2011; Zander et al. 2011; Mouritsen et al. 2011; Preston et al. 2012). Links identified from the literature were included in the current food web based on the predators’ ability to feed on small-sized organisms, such as ciliates and other protozoans, combined with known or measured size of the free-living parasitic stages of the parasite species in question, while also considering their typical habitat affiliation and behavior (i.e., Lafferty et al. 2006a, b; Orlofske et al. 2015; see also Koprivnikar et al. 2023). Five of these links (between stickleback/amphipods and three species of trematode cercariae) have later been confirmed in Takvatn by experimental studies (Born-Torrijos et al. 2020, 2021). Trematodes dominated the free-living parasite stage links with their free-living stages (miracidia and cercariae) contributing to 80% of this type of interaction. Predators of these free-living stages were dominated by chironomids and cladocerans, but a large range of organisms were identified as plausible predators.

Parasite-parasite links in this system (26 links) are represented by intraguild predation, which is prevalent among larval trematodes when they share a common host individual (typically snails) and furthermore share an infected organ (Kuris 1990; Lafferty et al. 1994). Trematodes were assumed to feed on each other based on common dominance hierarchies exhibited by other species of larval trematodes in alternative snail host species (Kuris 1990; Kuris and Lafferty 1994; Soldánová et al. 2012). In the current food web, five trematode species were identified as likely predators on 13 different trematode species. No other parasite-parasite links were observed.

### Subweb construction

Four subwebs including different sets of trophic interactions were created. The first subweb included only predator–prey links between free-living taxa, including predation, cannibalism, and detritivory. We then added all parasitic taxa to obtain the second web, which consisted of the predator–prey links as well as the parasite–host links, describing interactions between parasites and their free-living hosts via macroparasitism, trophic transmission, pathogen infection, and castration. This subweb included all free-living and parasitic nodes in the food web and was used to calculate the trophic level of each node, or taxa, and to provide a visual representation of the food web. The third subweb included free-living taxa preying upon free-living, non-feeding stages of parasites, such as fungi, cestodes, and trematodes, in addition to predator–prey and parasite-host interactions. These links could end in parasite infection for the free-living predator, or the parasite could merely be digested. This subweb was used to calculate the generality, as it is descriptive of the prey choice of each consumer taxa. The final subweb analyzed in this study included all the previously described interaction types as well as predator-parasite and parasite-parasite links. Accordingly, this web considers all possible predator–prey, parasite-host, predator-parasite, and parasite-parasite interactions.

These subwebs were then used to construct three versions of the food web: the pelagic food web (updated from Amundsen et al. 2009), the benthic food web, and the whole-lake food web. Each free-living node was assigned to the pelagic or benthic compartment based on their predominant habitat use (as determined through direct observations and literature describing “typical” habitat for species) and the predominant habitat of their most common prey items. Similarly, parasites were assigned based on the habitat that their obligatory hosts are found in. For example, all trematodes were assigned to the benthic compartment, as this is where their key hosts are infected, including their obligatory first hosts—mollusks. A few species found in both pelagic and benthic habitats were included in both compartments. These were typically higher trophic level species with high mobility (fishes and birds) and have been described as habitat-integrators (Vander Zanden and Vadeboncoeur 2002). The benthic web includes one node (representing water mites) that consists of both a parasitic life stage and a free-living life stage and is thus recognized as both parasitic and free-living. The free-living web only incorporates the free-living life stage of this node and the predator–prey interactions linked to that life stage, while the other subwebs incorporate all relevant links associated with this node, both parasitic and free-living. Finally, the nodes present in each compartment were combined into a single food web, representing the whole lake. This food web also accounts for all links occurring between the pelagic and benthic taxa.

### Food-web analyses

Life-stage nodes were aggregated to the species level as the lowest level of resolution (see Dunne et al. 2013; Morton and Lafferty 2022). Aggregation can mask the specialist trophic niches exhibited by a species at each stage of its life cycle and cause species to appear much more general in diet with phylogenetically discontinuous trophic niches (Dunne et al. 2013). However, aggregation allows comparison with previously published food-web analyses and can help reduce biases introduced from uneven sampling of species and their ontogenetic stages. Yet, the degree to which nodes are aggregated can differ between published analyses and often reflect system differences. For example, Takvatn has four nodes representing detritus, including terrestrial leaves and other terrestrial vegetation, while many other analyses aggregate all forms of detritus into just two nodes (Dunne et al. 2013). The analysis of Takvatn also includes eleven nodes representing phytoplankton, whereas the salt marsh analysis conducted by Lafferty et al. (2006b) includes just one phytoplankton node.

To allow further comparison to other food webs with parasites and address our objectives, ten key metrics which describe food-web structure were calculated for each of the three food webs (Table 1). We observed the number of nodes and links and calculated the number of trophic levels, linkage density, connectance, adjusted connectance (Lafferty et al. 2006a), mean degree, generality, and vulnerability for all subwebs. Generality and vulnerability distributions, as well as those across parasites and free-living taxa were also calculated for each food web and compared using *Z* tests. We did not compare generality for the web including concomitant and parasite–parasite links because these interactions do not contribute to the free-living predator’s or parasite’s diet or host breadth and do not factor into the movement of energy in a food web (Morton and Lafferty 2022).

To control for changes in topological metrics with food-web size, we compared parasite associated changes in topological metrics for each food web (pelagic, benthic and whole-lake) with simulated food webs of the same size and connectance (Williams and Martinez 2000). We used the niche web model in the *trophic* package (https://rdrr.io/github/jjborrelli/trophic/) following the methods reported in Morton and Lafferty (2022). Briefly, for each food-web assembly, we simulated 1000 networks with connectance (adjusted connectance was used for webs including parasites) and size matching the empirical food webs (Williams and Martinez 2000). For metrics that vary within the niche model, we calculated model error (ME) as the normalized difference between the median model value and the empirical vale to allow comparison between webs of different sizes (Williams and Martinez 2008). Positive ME indicates a metric is overrepresented in the empirical web relative to the simulated models, negative ME indicates underrepresentation in the empirical web, and |ME|> 1 indicates the empirical value is not within the most likely 95% model values (significantly different) (Williams and Martinez 2008). Model fits decline with increasing network size, particularly with webs > 100 nodes (Williams and Martinez 2008; Williams and Purves 2011; Dunne et al. 2013; Wood et al. 2015; Vinagre et al. 2019), so this caveat was considered in the interpretation of the results.

All analyses were completed in R Version 4.0.3 (R Core Team 2020) with the packages *igraph* (Csardi and Nepusz 2006), *NetIndices* (Kones et al. 2009), and *trophic* (https://rdrr.io/github/jjborrelli/trophic/). These topological metrics were used to make pairwise comparisons between each food web’s free-living web and web with parasites, as well as to compare the pelagic and benthic food webs with parasites. More attention was given to the pelagic and benthic analyses as these two compartments combined comprise the whole-lake analysis and including also the whole-lake analysis would thus be repetitive for some metrics.

## Results

### Food-web topology

Seven nodes, including the three fish species present in the lake (trout, charr, and stickleback), three duck species (*Melanitta nigra*, *Melanitta fusca*, and *Bucephala clangula*), and a cladoceran (*Polyphemus pediculus*), can be found in both the pelagic and benthic habitats of Takvatn, and so were represented in the analysis for both compartments. The pelagic compartment’s predator–prey subweb consisted of 37 taxa that were linked via 209 predator–prey trophic interactions across four trophic levels (Table 3; Figs. ESM.1a; 2a). The larger and more complex benthic compartment had a predator–prey subweb made up of 98 taxa, and their 1731 links were only spread across three trophic levels (Table 3; Figs. ESM.1b; 2b). While phytoplankton, rotifers, and crustacean zooplankton were the most common free-living taxonomic groups identified in the pelagic compartment, the most common groups found in the free-living benthic community were periphyton, cladocerans, insect larvae, bivalves, snails, and worms.

**Table 3:** Summary of calculated key topological food-web metrics for each of the four subwebs in the three compartments

Metric	Web	Predator–prey	Predator–prey + parasite-host (no predator-parasite + no parasite-parasite)	Predator–prey + parasite-host + predator-parasite (no parasite-parasite)	Predator–prey + parasite-host + predator-parasite + parasite-parasite
Nodes	Pelagic	37	53	53	**53**
	Benthic	98	128	128	**128**
	Whole-lake	128	174	174	**174**
Observed links	Pelagic	209	277	512	**512**
	Benthic	1731	1943	3244	**3270**
	Whole-lake	2017	2347	4250	**4276**
Trophic levels	Pelagic	4.4	**4.7**	9.2	9.2
	Benthic	3.8	**4.3**	8.8	9.3
	Whole-lake	4.0	**4.5**	10.4	10.6
Longest chain	Pelagic	3.0	3.0	4.0	**4.0**
	Benthic	6.0	5.0	5.0	**5.0**
	Whole-lake	7.0	6.0	5.0	**5.0**
Linkage density	Pelagic	5.65	5.23	9.66	**9.66**
	Benthic	17.66	15.18	25.34	**25.55**
	Whole-lake	15.76	13.49	24.43	**24.57**
Connectance	Pelagic	0.153	0.099	0.182	**0.182**
	Benthic	0.180	0.119	0.198	**0.200**
	Whole-lake	0.123	0.078	0.140	**0.141**
Adjusted connectance	Pelagic	–	0.141	0.201	–
	Benthic	–	0.155	0.210	–
	Whole-lake	–	0.105	0.151	–
Mean degree	Pelagic	11.30 (± 6.78)	10.45 (± 8.31)	19.32 (± 10.39)	**19.32 (± 10.39)**
	Benthic	35.33 (± 17.47)	30.36 (± 21.15)	50.69 (± 26.57)	**51.09 (± 26.90)**
	Whole-lake	31.52 (± 19.03)	26.98 (± 21.53)	48.85 (± 30.36)	**49.15 (± 30.68)**
Mean generality	Pelagic	5.65 (± 5.65)	5.23 (± 5.09)	**7.70 (± 8.63)**^a^	9.66 (± 9.27)
	Benthic	17.66 (± 19.06)	15.18 (± 17.64)	**22.36 (± 21.84)**^a^	25.55 (± 25.80)
	Whole-lake	15.76 (± 18.46)	13.49 (± 16.67)	**20.38 (± 21.88)**^a^	24.57 (± 27.20)
Mean vulnerability	Pelagic	5.65 (± 4.18)	5.23 (± 5.59)	9.66 (± 6.59)	**9.66 (± 6.59)**
	Benthic	17.66 (± 11.04)	15.18 (± 11.96)	25.34 (± 17.48)	**25.55 (± 17.91)**
	Whole-lake	15.76 (± 10.82)	13.49 (± 11.73)	24.42 (± 18.71)	**24.57 (± 19.09)**

Bold values denote those used for metric comparisons among compartments

^a^Generality for the predator–prey + parasite-host + predator-parasite subwebs was calculated without concomitant predation because these interactions do not contribute to the free-living predator’s host breadth and do not factor into the movement of energy in a food web (Morton and Lafferty 2022)

**Fig. 1:**
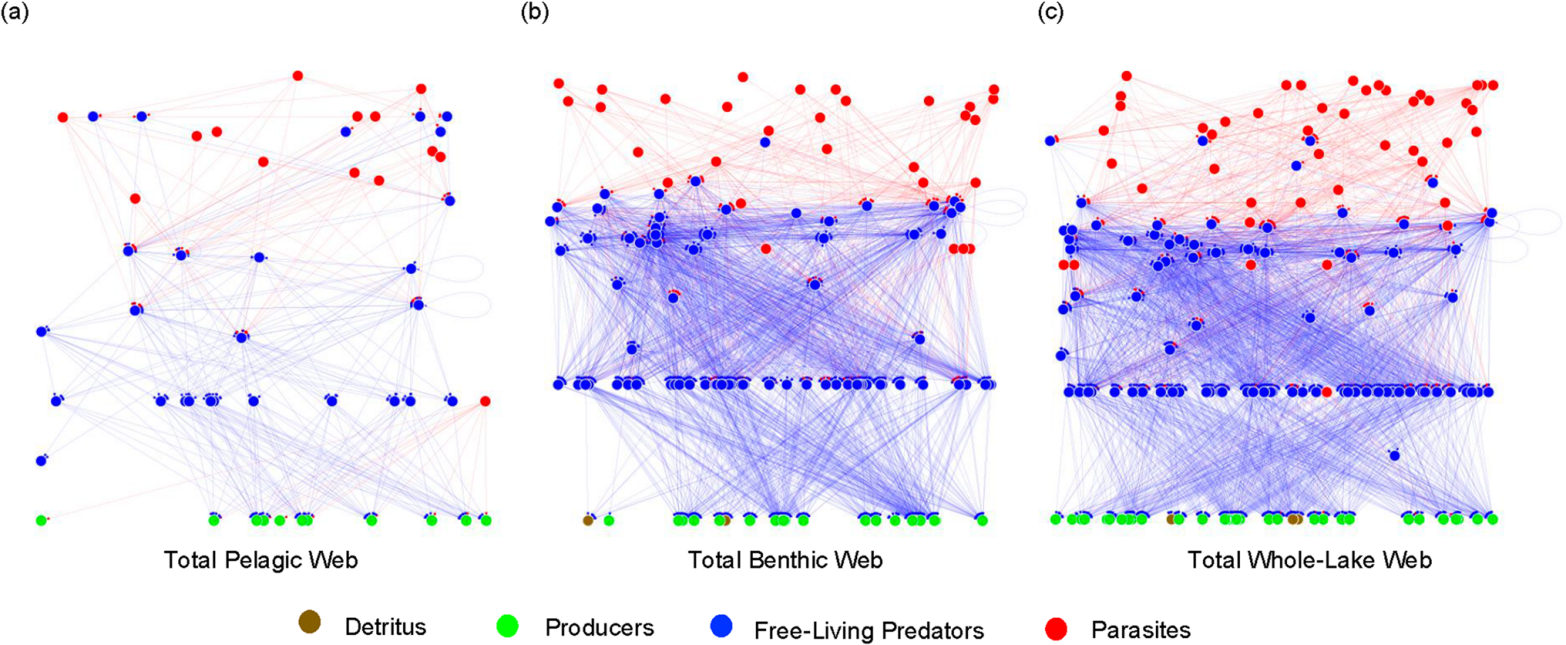
Food web of Takvatn’s (**a**) total pelagic web, (**b**) total benthic web, and (**c**) total whole-lake web and their respective links. The nodes representing detritus and other non-living taxa are depicted in brown, the producers are depicted in green, and all other free-living taxa are depicted in blue. The links between these free-living nodes are blue. All parasites are depicted in red and their respective parasitizing links are also red

**Fig. 2:**
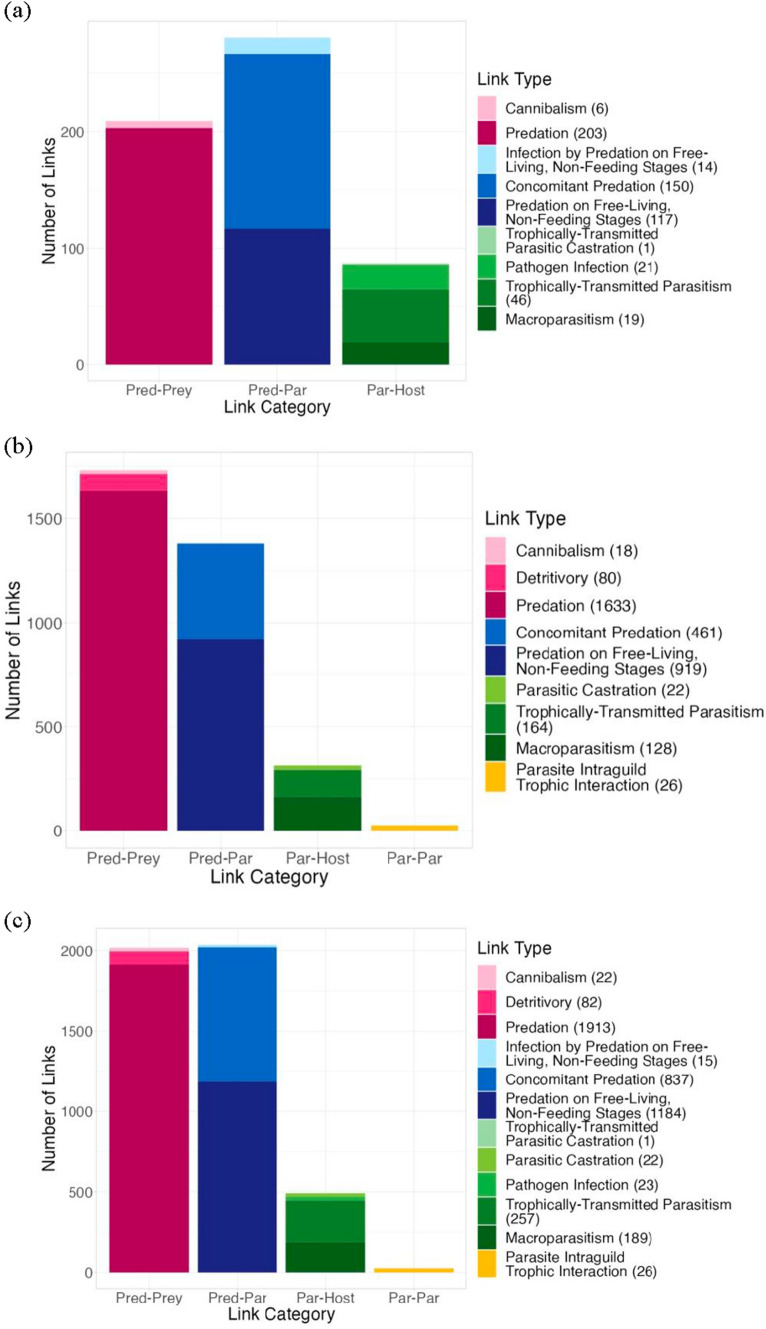
The number of links in the (**a**) total pelagic compartment, (**b**) total benthic compartment, and (**c**) total whole-lake web. Each of the four link categories (predator–prey in pink, predator-parasite in blue, parasite-host in green, and parasite-parasite in yellow) is subdivided into its corresponding link types (twelve total). The number of links observed in each link type is presented in parentheses next to the link type name. The pelagic compartment did not contain any parasite-parasite links as only trematodes exhibited this link category, and all trematodes were allocated to the benthic compartment. The number of links in the free-living webs of each web version are represented by the number of predator–prey links present in each total web

The addition of parasites in the pelagic web increased the number of taxa present to 53, including 12 basal taxa (primarily phytoplankton), 27 free-living predators, and 14 parasites (Table 3; Fig. 1a). The difference in the number of basal taxa relative to the free-living pelagic web is due to two inedible phytoplankton species (*Planktothrix mougeotii* and *Tabellaria flocculosa v. geniculata*), whose only connection to the rest of the web is that they are parasitized by chytrid fungi and thus were not included in the predator–prey web. In contrast, the total benthic web with its higher level of complexity consisted of 128 nodes and included 23 basal taxa (including detritus, periphyton, and macrophytes), 75 free-living predators, and 31 parasites (Table 3; Fig. 1b). While the benthic compartment has over double the number of taxa as the pelagic compartment, the number of links present in the benthic compartment is over six times the number of links observed in the pelagic (Table 3; Fig. 2a, b). Additionally, parasites made up a similar proportion of each web’s taxa (26% in the pelagic and 24% in the benthic), however, the pelagic parasites were involved in 59% of all links in the pelagic web and the benthic parasitic links made up 48% of all trophic interactions in the benthic web (Fig. 2a, b). In the pelagic compartment, cestodes were by far the most common parasite taxa, whereas trematodes dominated the parasite community in the benthic compartment followed by gregarines. Additionally, including parasites had little effect on the number of trophic levels observed in all three webs (Table 3).

Similar results were observed in the whole-lake food web. The free-living whole-lake food web was made up of 128 nodes spread across four trophic levels connected via 2017 links (Table 3; Figs. ESM.1c; 2c). Including parasites increased the number of nodes, links, and trophic levels. The whole-lake web with parasites consisted of 174 nodes connected by 4276 links dispersed across four trophic levels and comprised of 35 basal taxa, 95 free-living consumers, and 45 parasitic taxa (Table 3; Figs. 1c; 2c).

### Chain length

**Fig. 3:**
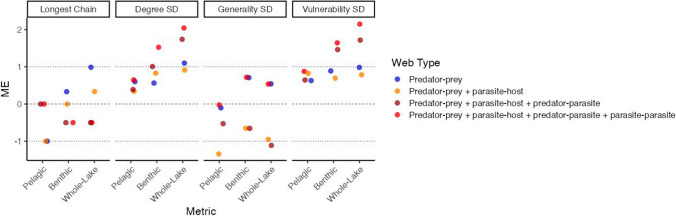
Niche model errors (ME) for four network structure properties for four web versions of the three Takvatn compartments. MEs >|1| indicate a significant difference between the empirical value and niche model prediction (1000 simulated networks). Negative MEs indicate the empirical value was less than model predicted value; positive MEs indicate the empirical value was greater than model predicted value

The effects of adding parasites to the food web on chain length differed between the pelagic and benthic compartments. Adding parasites to the pelagic food web increased longest chain length from three to four species (Table 3). However, adding parasites to the benthic food web decreased chain length from six to five species. This decrease was also observed in the whole-lake food web, where adding the parasite-host subweb decreased longest chain length from seven to six and adding the predator-parasite subweb further decreased longest chain length to five. Longest chain lengths in all web versions were statistically similar to expectations based on network size, and therefore the observed changes in chain length with the addition of parasites were due to adding species rather than adding parasites specifically (Fig. 3). The free-living webs tended to have the largest model errors in all three compartments (marginally shorter than predicted in the pelagic compartment, and marginally longer than predicted in the whole-lake compartment).

### Linkage density

Including parasites in the analysis substantially increased the number of observed links, which served to increase linkage density from the free-living webs to the webs with parasites in all three web comparisons (Table 3; pelagic compartment: free-living = 5.65 vs with parasites = 9.66; benthic compartment: free-living = 17.66 vs with parasites = 25.55; whole-lake web: free-living = 15.76 vs with parasites = 24.57). Additionally, when comparing the linkage density of free-living and parasite taxa in each of the three subwebs with all parasite links included, on average, parasites were always slightly more connected than their free-living counterparts (pelagic compartment: free-living = 9.24 vs parasites = 10.82; benthic compartment: free-living = 25.38 vs parasites = 25.85; whole-lake web: free-living = 24.10 vs parasites = 25.81).

### Unadjusted and adjusted connectance

Adding parasites to the food webs increased both unadjusted and adjusted connectance. Yet, while the whole-lake web had a smaller connectance than the smaller pelagic web, the benthic web had the largest connectance. The additional links provided by parasites increased unadjusted connectance of the webs with parasites in the pelagic compartment (free-living = 0.153 vs with parasites = 0.182), benthic compartment (free-living = 0.180 vs with parasites = 0.200), and whole-lake food web (free-living = 0.123 vs with parasites = 0.141) (Table 3). However, when comparing the predator–prey subweb with the predator–prey + parasite-host web, unadjusted connectance decreased slightly in each of the three compartments. This was rectified when adjusting the denominator in the calculation for connectance to account for the number of observed links more accurately in these subwebs, and adjusted connectance also increased with the addition of parasites (Table 3).

### Generality

**Fig. 4:**
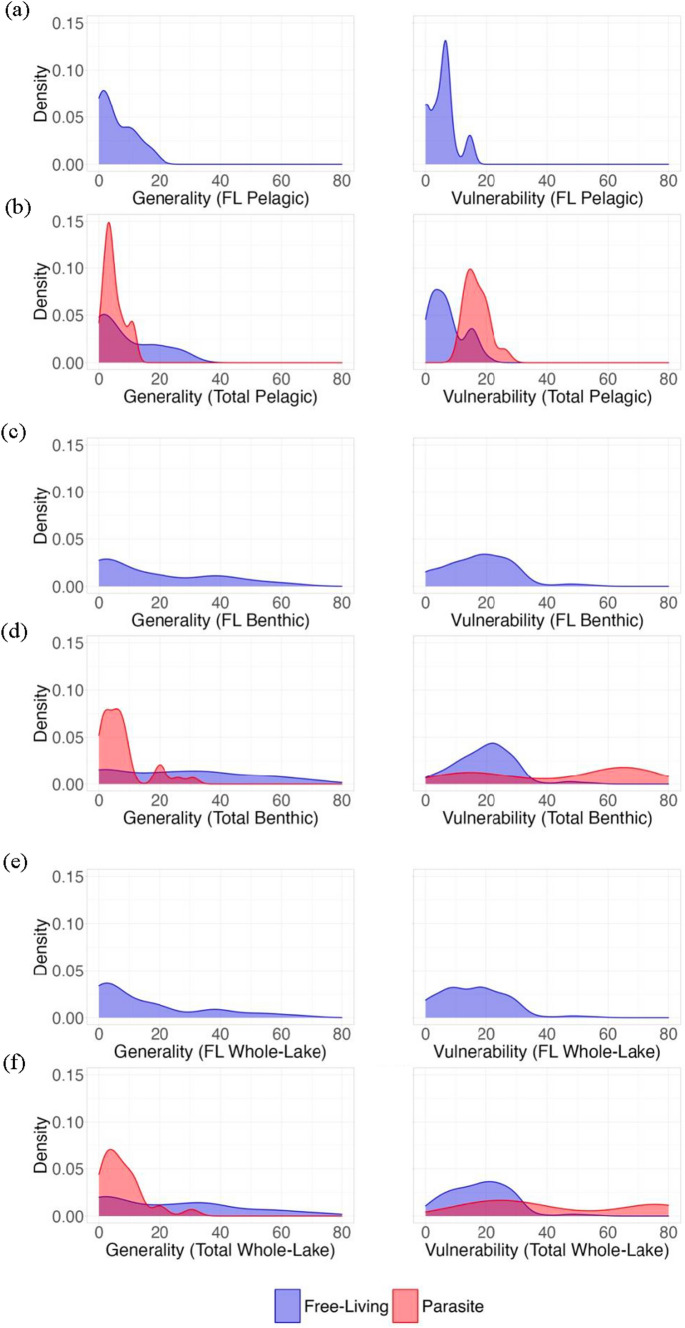
Density plots depicting generality and vulnerability distributions of (**a**) the free-living pelagic web and (**b**) the total pelagic web, (**c**) the free-living benthic web and (**d**) the total benthic web, and (**e**) the free-living whole-lake web and (**f**) the total whole-lake web. In each panel, generality and vulnerability for each web are further sub-divided into free-living taxa generality and vulnerability densities and parasite taxa generality and vulnerability densities. Free-living taxa values are represented in blue and parasite taxa values are represented in red

Each compartment had nominally higher generality when parasites were included (Table 4). However, free-living taxa generality was only increased in the benthic and whole-lake webs after adding parasites. Additionally, in both the pelagic and benthic compartments, there was no difference in food-web generality between the predator–prey subweb and the food webs with parasites (Table 4; Fig. 4a–d). This is likely because including parasites in the pelagic compartment did not lead to an increase in generality of free-living taxa between the predator–prey subweb and the pelagic web with parasites. However, this contrasts the benthic compartment in which including parasites in the analysis increased the generality of free-living taxa (Table 4; Fig. 4c, d). Free-living taxa in both compartments exhibited a larger diet breadth than their parasitic counterparts’ host breadth (Table 4; Fig. 4b, d). In the whole-lake food web, including parasites led to significant increases in generality (Table 4; Fig. 4e, f). In this web, not only did free-living taxa generality increase in the analysis with parasites, but so too did the entire food-web generality.

**Table 4:** Results from *Z* test comparisons of generality and vulnerability in each of the three versions of the food web

	Free-living	Parasite	Pelagic web		Benthic web		Whole-lake web	
			*Z* score	*P* value	*Z* score	*P* value	*Z* score	*P* value
Generality	Generality of subweb without parasites	Generality of subweb with parasites	1.3606	0.17	1.7222	0.085	1.9862	**0.047**
	Generality of free-living taxa	Generality of parasite taxa	− 2.1663	**0.030**	− 7.2457	**4.30e−13**	− 7.2787	**3.73e−13**
	Generality of free-living taxa in subweb without parasites	Generality of free-living taxa in subweb with parasites	1.6943	0.090	3.1395	**0.0017**	3.4248	**0.00062**
Vulnerability	Vulnerability of subweb without parasites	Vulnerability of subweb with parasites	3.5267	**0.00042**	4.0721	**4.66e-05**	5.0826	**3.72e−07**
	Vulnerability of free-living taxa	Vulnerability of parasite taxa	7.2142	**5.42e−13**	5.0643	**4.10e−07**	6.3731	**1.85e−10**
	Vulnerability of free-living taxa in subweb without parasites	Vulnerability of free-living taxa in subweb with parasites	1.3174	0.19	1.4569	0.15	1.7639	0.078

Positive *Z* scores indicate that the parasite value is larger than the free-living. Negative *Z* scores indicate that the free-living value is larger than the parasite value. Bold *P* values denote significant differences

Only two web versions were significantly different from the model predictions of generality with the addition of parasites: the pelagic predator–prey + parasite-host subweb and whole-lake subweb that excluded parasite-parasite interactions (Fig. 3). In these webs, the standard deviation of generality was lower in the empirical food web than the simulated webs, indicating that the range of generality was smaller than predicted based on web size alone for both webs, and that the increase in generality for these two webs was a response specifically to the addition of parasites in these web versions.

### Vulnerability

In each of the three webs, parasites increased food-web vulnerability (*p* < 0.05; Table 4; Fig. 4). However, this was not the result of an increase in free-living vulnerability. While including parasites did add enemies for many free-living taxa, this only increased the mean vulnerability of free-living taxa nominally and was not enough to change the vulnerability distributions in a statistically significant way (*p* < 0.05; Table 4; Fig. 4). Additionally, parasites were more vulnerable than their free-living counterparts in each of the total webs (Table 4; Fig. 4), which apparently is the key contributor to the increased food-web vulnerability in the food webs that included parasites.

In the three subwebs including all parasite interactions, the most vulnerable taxa consisted solely of parasites. While the free-living pelagic web’s most vulnerable taxa mostly consisted of phytoplankton taxa and the stickleback, all the most vulnerable taxa in the total pelagic web were parasites, specifically a species of oomycote and numerous cestodes. Similarly, in the total whole-lake web and total benthic compartment, the most vulnerable taxa were all parasitic trematodes, whereas the free-living web’s most vulnerable list was made up of producers and chironomids.

While in the pelagic compartment, most parasites were vulnerable to many predators (Fig. 4b), parasites in the benthic and whole-lake food webs had either few or many predators (Fig. 4d, f). Most free-living taxa were, in contrast, vulnerable to predation or parasitism from a relatively low number of enemies. In each food web, there were several taxa that were not vulnerable to any other taxa. In the free-living pelagic food web, the seven pelagic bird taxa were not vulnerable to predation from any other taxa in the free-living pelagic web. However, with the inclusion of parasites, Takvatn’s pelagic bird taxa became vulnerable to parasitism, thus removing them from the top predator position in the pelagic food web with parasites. Similarly, in the benthic food web, all seven benthic bird taxa, the mink (*N. vison*), and a leech (*Glossiphonia complanata*) had no trophic vulnerabilities in the free-living web, whereas in the total benthic web only the leech remained resilient against parasitism.

In the benthic and whole-lake subwebs that included predator-parasite interactions and the subwebs that included all parasite interactions, the standard deviation of vulnerability was significantly larger than expected based on network size alone (Fig. 3), indicating that the vulnerability measured of these subwebs is a result of the addition of parasites specifically, rather than merely the addition of taxa. This was most pronounced for the whole-lake compartment, whereas this effect was not observed in the pelagic compartment.

## Discussion

Incorporating parasites into Takvatn’s food webs allows for further insight into the structure and functioning of the total food web and ultimately the whole ecosystem. The observed increase in the number of trophic levels, linkage density, connectance, and mean generality and vulnerability corroborates the findings of other studies that have demonstrated that including parasites in food-web analyses increases most topological metrics (Lafferty et al. 2006b; Hernandez and Sukhdeo 2008; Preston et al. 2014; McLaughlin 2018) and agrees with our predictions. The sensitivity of the metrics to the types of links included in each food-web version and the generality of these findings as seen from similar studies in other systems (e.g., Lafferty et al. 2006b; Hernandez and Sukhdeo 2008; Preston et al. 2014; McLaughlin 2018), point to the important role that parasites play in trophic interactions within an ecosystem. This is further supported by our analysis of the benthic and pelagic food-web compartments in Takvatn, which demonstrates that the distinctive parasite communities of these highly contrasting subwebs, i.e., the benthic food web with its highly general and simultaneously vulnerable trematodes and the pelagic food web with its numerous cestodes, alter the food-web metrics in a similar way. Consequently, the same effects of the inclusion of parasites in food-web analysis are seen both from very dissimilar ecosystems covering marine, freshwater, and terrestrial biotopes, and from two highly contrasting habitats within a lacustrine ecosystem.

Some metrics did not change in the way we predicted they would with the addition of parasites. Chain length was expected to increase when adding parasites because they introduce consumer pressure to free-living taxa and increased the number of trophic levels, and this was observed in the pelagic compartment. However, the longest chain shortened in the benthic and whole-lake webs, irrespective of what type of parasite links were included. This may be a consequence of node aggregation as aggregating life stages into lower resolution taxonomic groups makes predators of a single life stage become predators of a whole taxonomic group, thus masking the specialist trophic niches of the predator, and shortening the food chain. Yet, while there were nominal changes between empirical and model predicted values for chain length, these observed differences were not significant and were a consequence of network size more so than of the specific characteristics of parasites, indicating that parasites have little effect on chain length.

As predicted, connectance increased with the inclusion of parasites in all three web comparisons, similar to the findings of several previous analyses comparing food webs with and without parasites across numerous and highly contrasting types of ecosystems, including salt marsh (Lafferty et al. 2006b), stream (Hernandez and Sukhdeo 2008), pond (Preston et al. 2014), atoll (McLaughlin 2018), and harbor and estuary systems (Dunne et al. 2013). Morton and Lafferty (2022), in contrast, observed a decrease in connectance with the addition of parasites in their kelp forest food web, suggesting that this was due to a combined effect of the large food-web size, the relatively high level of specialization exhibited by parasites, and the lack of intraguild predation and predation on free-living stages of parasites in this system. Contrary to our conditions, the benthic web had the largest connectance, for reasons likely related to the generality and vulnerability of this compartment’s main parasite taxa—the trematodes.

While we expected an increase in generality with the addition of parasites, especially when considering the effect of aggregating parasite life stages, mean generality was not observed to significantly differ between the free-living and total webs of the pelagic and benthic compartments, although it was nominally greater when parasites were included and did in fact differ significantly between the whole-lake subwebs. Even though the inclusion of parasites introduces a new trophic interaction category in which the parasites are consumers (i.e., the parasite-host links), these links did not statistically increase the overall generality of the food web. Rather, an increase in generality was only observed after also considering the predator-parasite and parasite-parasite links, a result that was also observed in a salt marsh system (Lafferty et al. 2006b). Furthermore, the inclusion of parasites also served to increase free-living generality in the benthic compartment, as predicted, but not in the pelagic. The pelagic food web contains many specialized parasites that infect four or less free-living taxa (e.g., the specialist copepod *Salmincola edwardsii* only infects charr), while free-living taxa in the pelagic web prey on an average of nine taxa. The benthic compartment had higher generality, largely due to the presence of the general trematodes. The trematodes present in Takvatn’s benthic compartment parasitize as many as 26 free-living hosts, which is a higher generality than almost half of the benthic free-living taxa. In addition, the numerous trematode cercariae present in Takvatn serve as prey for many free-living predators, so free-living generality was also enhanced in the benthic compartment.

While the pelagic food web also contained predation on free-living parasite stages, they were not numerous enough to increase the pelagic free-living taxa’s generality in a significant way. It should be noted that the inclusion of concomitant links could potentially have increased the generality of the total webs, and in fact we did observe a higher generality in the subweb that included this link type in the generality calculation (pelagic compartment: 9.66, benthic compartment: 25.55, whole-lake: 24.57). However, these links were not included in the calculation of the generality of free-living taxa because predators do not gain significant energy from consuming parasites within their hosts (relative to the free-living prey serving as host) and this consumption therefore has little effect on the web’s flow of energy to predators (Morton and Lafferty 2022). Additionally, our decision to aggregate life stages into species inflates the generality of trophically transmitted parasites and masks their specialistic trophic niches. However, in each of the webs, the free-living taxa were much more general than their parasitic counterparts. Furthermore, the standard deviation of generality was only smaller than predicted based on network size in two webs with parasites, the whole-lake subweb without concomitant predation and parasite-parasite links and the pelagic web with predator–prey and parasite-host links. Therefore, unlike with chain length, in these webs, parasites were responsible for the lower-than-expected generality, likely because the excluded link types greatly increase generality among taxa.

In contrast to food-web generality, the addition of parasites to Takvatn’s trophic food webs more consistently increased vulnerability, similar to findings from other systems (Lafferty et al. 2006a; Dunne et al. 2013; McLaughlin 2018; Morton and Lafferty 2022), and we observed increased vulnerability with the addition of parasites in all three webs. However, contrary to our predictions, this increase in vulnerability was not the result of an increase in free-living vulnerability. By definition, parasite-host links introduced new enemies for the free-living taxa, thereby increasing some taxa’s vulnerability. This was especially true for the amphipod, fishes, and birds. Additionally, each predator–prey interaction provides an opportunity for concomitant predation on parasites (death via consumption by unsuitable hosts, Johnson et al. 2010; Thieltges et al. 2013). The webs which included predator-parasite interactions contained extensive concomitant links and higher overall vulnerability. Additionally, 42–58% of links in all three webs with parasites were categorized as predator-parasite, while only 7–12% of the links were categorized as parasite-host. Therefore, parasite vulnerability was much greater than their generality, while the diet breadth and number of enemies of free-living taxa remained fairly consistent with and without parasites. This contributed to the overall increase in vulnerability in the food webs including parasites relative to the free-living webs. Furthermore, the addition of parasites increased the standard deviation of vulnerability more than predicted by network size in the benthic and whole-lake subwebs that included predator-parasite and parasite-parasite links, which is likely due to the life history of trematodes and the vulnerability they introduce to a food web.

Trematodes made a particularly strong contribution to the observed increase in connectance for the benthic and whole-lake webs in the Takvatn system. They were among the most vulnerable taxa as well as the most general parasites due to their complex life cycle, in which cercariae are released from their first intermediate hosts, typically *R. balthica* in Takvatn, and seek out a suitable second intermediate host (Orlofske et al. 2015; McKee et al. 2020). Cercariae can be very abundant in lake ecosystems and offer a glycogen- and lipid-rich food source for many non-host taxa (McKee et al. 2020). The complex life history of trematodes introduces multiple different link types to food webs.

While parasitizing their first intermediate host, trematodes introduce parasite-parasite interactions as larval trematodes compete for space within a common snail host (Kuris 1990; Lafferty et al. 1994). This results in intraguild predation as one trematode preys upon the other to eliminate the competition and utilize the snail’s resources, an interaction that is rare among free-living taxa (Kuris 1990; Lafferty et al. 1994). This link category has previously been reported from marine food webs and has been noted for its significance when considering the impacts of parasites, and specifically trematodes, on food-web structure and connectance (Lafferty et al. 2006b). In Takvatn, five of the trematode taxa contribute to a total of 26 parasite-parasite trophic interactions. Intraguild predation not only increased the trematodes’ vulnerability, but also the mean vulnerability of the benthic and total whole-lake. Moreover, when cercarial stages leave the snail to seek their next host, they are consumed by free-living predators, introducing many predator-parasite links (see also Born-Torrijos et al. 2020, 2021). During the trematodes’ free-living life stage they may become prey to pelagic zooplankton. However, we expect that these interactions do not reflect true pelagic trophic links but are rather occurring closer to shore within the boundary between the benthic and pelagic habitats. Additionally, trematodes do not infect any truly pelagic taxa, just fishes, which are most likely infected in the benthic, further supporting our decision to only include trematodes in the benthic compartment and not the pelagic compartment for this analysis. However, over 80% of the predation on free-living parasite stages in the whole-lake web involved cercariae. These trophic interactions drastically increased the vulnerability of trematodes and resulted in an increase in the total webs’ vulnerability as well, thus serving to increase connectance in the benthic and whole-lake food webs. Such disproportionate network effects due to the trematode’s complex life cycle were also observed in the similarly trematode-dominated salt marsh food web (Lafferty et al. 2006b). Even in such fundamentally different ecosystems (a subarctic lake vs a temperate marine environment), the trematodes, with their complex life cycles and multiple host species, are driving the changes seen in connectance, generality, and vulnerability when including parasites in food-web analyses.

Unlike the benthic compartment, which was dominated by the very general and vulnerable trematodes, the pelagic compartment was dominated by cestodes, which as expected were less vulnerable to free-living predation. Cestodes also have a free-living stage, coracidium, which only infect copepods and are vulnerable to concomitant predation. Additionally, cestodes use paratenic hosts, with stickleback being the key paratenic host in Takvatn (Henriksen et al. 2016; Kuhn et al. 2016a). These hosts do not serve to further the development of a parasite, but rather aid in the transmission of that parasite and bridge trophic gaps within a web through parasitic infections (Braicovich et al. 2016). Even with these two distinct parasite life history strategies in the pelagic and benthic compartments, the pelagic cestodes and benthic trematodes had very similar effects on their respective food webs when addressing the subweb that does not contain predator-parasite or parasite-parasite links.

Food-web connectance was predicted to be lower for the benthic web than for the smaller pelagic food web as connectance typically decreases with increasing food-web size (Riede et al. 2010). In support of this prediction, connectance was lowest in the whole-lake webs. However, in disagreement with our prediction, the benthic and pelagic compartments had similar connectance when parasites were included, with the benthic web having a slightly higher connectance. Even in the free-living subwebs, connectance was higher in the benthic when compared with the pelagic web. It has been suggested that a food-web’s connectance depends on the life history of its parasites (Lafferty et al. 2006b) and that parasites with higher host specificity contribute to a lower connectance (Memmott et al. 2000). For connectance to increase as food-web size increases, link density must increase exponentially as species are added to the network. Taxa in the benthic compartment were much more connected than those in the pelagic, as exhibited by its much larger (nearly tripled) linkage density. This trend was also observed when comparing linkage densities of the benthic and pelagic parasites and the benthic and pelagic free-living taxa. Furthermore, benthic parasites in Takvatn exhibited twice the diet breath and had more than twice as many enemies as their pelagic counterparts, thereby increasing the connectance of the benthic food web. This compartment contained almost five times as many predator-parasite links, mostly due to the presence of trematode cercariae, greatly increasing the parasites’ mean vulnerability and thereby also the mean vulnerability of the benthic compartment. Additionally, the benthic free-living taxa had over three times as many prey taxa as the pelagic free-living taxa and nearly three times as many predators. Together, these factors ultimately contributed to the high connectance observed in the benthic compartment.

Our results suggest potentially large impacts of parasites on food-web topology and ultimately ecosystem structure, functioning, and stability. Not only did we increase the size of each of the webs by adding parasites to its species list, but we also added the benthic compartment to gain a better picture of how the whole system is structured. The structural alterations observed here with the addition of taxa, and specifically parasites, likely serve to affect the system’s stability as well. Parasites, especially parasites with complex life cycles, have been claimed to reduce food-web robustness (Lafferty and Kuris 2009a; Lafferty 2012). Such results are easier to identify when nodes are not aggregated as they were in the current study. Even though parasites increased connectance in this analysis, thereby increasing the robustness of the food web, we hypothesize that parasites would decrease food-web robustness in Takvatn when considering how trematode-dominant the system is. In this analysis, trematodes disproportionately increased the connectance of the web, thereby increasing stability, yet when represented at the life-stage level they are much more sensitive to secondary extinction, which leads to decreases in stability (Lafferty and Kuris 2009a). These secondary extinctions can have serious consequences for the population size of the free-living hosts parasitized by trematodes, which could further encourage trophic cascades in the ecosystem benefitting or harming other free-living species as well (Lafferty and Kuris 2009b). Therefore, parasites can be useful bioindicators of degradation and recovery to assess the health of an ecosystem (Lafferty 2012). However, when considering food-web stability and robustness, attention must be given to the inter-compartmental links between the pelagic and benthic habitats provided by parasites, as they are likely to provide additional network stability. Further exploration is necessary in respect to the outcome of food-web stability with the addition of parasites, and special care must be taken when deciding what level of node resolution is reported. Additionally, the assumptions made when constructing links must also be carefully considered as we remind the reader that like most other food web constructions, some of the interactions in this food web have been inferred, and a degree of caution must thus be taken when interpreting the results.

The findings from the current study demonstrate how the inclusion of parasites alter the topology and dynamics of the food web, and thereby the understanding of the entire ecosystem due to their unique characteristics as infectious agents. When comparing the pelagic and benthic compartments in a subarctic lake system, even with highly contrasting free-living and parasite species compositions, we observed similar food-web patterns and responses demonstrated by increases in linkage density, connectance, generality, and vulnerability after controlling for network size. Such results are attributable to the parasites’ life history strategies and the types of trophic interactions they introduce to the food web. However, the benthic compartment had larger differences in free-living and parasite taxa generality and vulnerability than the pelagic compartment. Trematodes are important contributors to these differences, as these benthic parasites are more vulnerable and general than other parasite taxa. Similar results were also observed in the whole-lake food web that combined both the pelagic and benthic compartments, making this subarctic lake comparable to most other food-web analyses conducted in various other ecosystems and climates (Lafferty et al. 2006b; Hernandez and Sukhdeo 2008; Preston et al. 2014; McLaughlin 2018). Consequently, our results confirm that parasites, and especially trophically-transmitted species, play a large role in the structuring and functioning of ecosystems, via altering energy flows and affecting trophic cascades, and should thus be integrated into analyses of food webs.

### Supplementary Information

Below is the link to the electronic supplementary material.

Supplementary file1 (PDF 312 KB)

## Data Availability

The datasets used and/or analyzed during the current study are available from the corresponding author on reasonable request. Many supporting datasets from other food webs and analyses have been cited throughout the text.

## Code availability

The code used during the current study are available from the corresponding author on reasonable request.

## References

[CR1] Amundsen P-A, Klemetsen A (1988). Diet, gastric evacuation rates and food consumption in a stunted population of Arctic charr, *Salvelinus alpinus* L., in Takvatn, northern Norway. J Fish Biol.

[CR2] Amundsen PA, Kristoffersen R, Knudsen R, Klemetsen A (1997). Infection of *Salmincola edwardsii* (Copepoda: Lernaeopodidae) in an age-structured population of Arctic charr—a long-term study. J Fish Biol.

[CR3] Amundsen P-A, Knudsen R, Klemetsen A (2007) Intraspecific competition and density dependence of food consumption and growth in Arctic charr. J Anim Ecol:149–158 10.1111/j.1365-2656.2006.01179.x 17184363

[CR4] Amundsen P-A, Lafferty KD, Knudsen R, Primicerio R, Klemetsen A, Kuris AM (2009). Food web topology and parasites in the pelagic zone of a subarctic lake. J Anim Ecol.

[CR5] Amundsen P-A, Lafferty KD, Knudsen R, Primicerio R, Kristoffersen R, Klemetsen A, Kuris AM (2013). New parasites and predators follow the introduction of two fish species to a subarctic lake: implications for food-web structure and functioning. Oecologia.

[CR6] Amundsen P-A, Primicerio R, Smalås A, Henriksen EH, Knudsen R, Kristoffersen R, Klemetsen A (2019). Long-term ecological studies in northern lakes—challenges, experiences, and accomplishments. Limnol Oceanogr.

[CR7] Anderson TK, Sukhdeo MV (2011). Host centrality in food web networks determines parasite diversity. PLoS ONE.

[CR8] Baia RRJ, Florentino AC, Silva LMA, Tavares-Dias M (2018). Patterns of the parasite communities in a fish assemblage of a river in the Brazilian Amazon region. Acta Parasitol.

[CR9] Banerji A, Duncan AB, Griffin JS, Humphries S, Petchey OL, Kaltz O (2015). Density-and trait-mediated effects of a parasite and a predator in a tri-trophic food web. J Anim Ecol.

[CR10] Barber I, Scharsack J (2009). The three-spined stickleback-*Schistocephalus solidus* system: an experimental model for investigating host-parasite interactions in fish. Parasitology.

[CR11] Beaman M, Madge S (2010) The handbook of bird identification: for Europe and the western Palearctic. A&C Black

[CR12] Born-Torrijos A, Paterson RA, van Beest GS, Schwelm J, Vyhlídalová T, Henriksen EH, Knudsen R, Kristoffersen R, Amundsen P-A, Soldánová M (2020). Temperature does not influence functional response of amphipods consuming different trematode prey. Parasitol Res.

[CR13] Born-Torrijos A, Paterson RA, van Beest GS, Vyhlídalová T, Henriksen EH, Knudsen R, Kristoffersen R, Amundsen PA, Soldánová M (2021). Cercarial behaviour alters the consumer functional response of three-spined sticklebacks. J Anim Ecol.

[CR14] Braicovich PE, Kuhn JA, Amundsen P-A, Marcogliese DJ (2016). Three-spined stickleback *Gasterosteus aculeatus*, as a possible paratenic host for salmonid nematodes in a subarctic lake. Parasitol Res.

[CR15] Brittain JE (1978). The Ephemeroptera of Øvre Heimdalsvatn. Ecography.

[CR16] Brittain JE (1978). The Mollusca of the exposed zone of Øvre Heimdalsvatn. Ecography.

[CR17] Britton JR, Andreou D (2016). Parasitism as a driver of trophic niche specialisation. Trends Parasitol.

[CR18] Byers JE (2009). Including parasites in food webs. Trends Parasitol.

[CR19] Campbell R, Haedrich R, Munroe T (1980). Parasitism and ecological relationships among deep-sea benthic fishes. Mar Biol.

[CR20] Cohen JE (1978). Food webs and niche space.

[CR21] Csardi G, Nepusz T (2006). The igraph software package for complex network research. InterJ Complex Sys.

[CR22] Dobson AP (1988). The population biology of parasite-induced changes in host behavior. Q Rev Biol.

[CR23] Dunne JA, Lafferty KD, Dobson AP, Hechinger RF, Kuris AM, Martinez ND, McLaughlin JP, Mouritsen KN, Poulin R, Reise K (2013). Parasites affect food web structure primarily through increased diversity and complexity. PLoS Biol.

[CR24] Frainer A, Johansen KMS, Siwertsson A, Mousavi SA, Brittain JE, Klemetsen A, Knudsen R, Amundsen P-A (2016). Variation in functional trait composition of benthic invertebrates across depths and seasons in a subarctic lake. Fundam Appl Limnol.

[CR25] Frainer A, McKie BG, Amundsen P-A, Knudsen R, Lafferty KD (2018). Parasitism and the biodiversity-functioning relationship. Trends Ecol Evol.

[CR26] Guo F, Kainz MJ, Sheldon F, Bunn SE (2016). The importance of high-quality algal food sources in stream food webs–current status and future perspectives. Freshw Biol.

[CR27] Hatcher MJ, Dick JT, Dunn AM (2006). How parasites affect interactions between competitors and predators. Ecol Lett.

[CR28] Heins DC, Baker JA (2008) The stickleback-*Schistocephalus* host-parasite system as a model for understanding the effect of a macroparasite on host reproduction. Behaviour:625–645

[CR29] Henriksen EH, Knudsen R, Kristoffersen R, Kuris AM, Lafferty KD, Siwertsson A, Amundsen P-A (2016). Ontogenetic dynamics of infection with *Diphyllobothrium* spp. cestodes in sympatric Arctic charr *Salvelinus alpinus* (L.) and brown trout *Salmo trutta* L. Hydrobiologia.

[CR30] Henriksen EH, Frainer A, Knudsen R, Kristoffersen R, Kuris AM, Lafferty KD, Amundsen P-A (2019). Fish culling reduces tapeworm burden in Arctic charr by increasing parasite mortality rather than by reducing density-dependent transmission. J Appl Ecol.

[CR31] Hernandez AD, Sukhdeo MV (2008). Parasites alter the topology of a stream food web across seasons. Oecologia.

[CR32] Huxham M, Raffaelli D, Pike A (1995). Parasites and food web patterns. J Anim Ecol.

[CR33] Johnson PT, Dobson A, Lafferty KD, Marcogliese DJ, Memmott J, Orlofske SA, Poulin R, Thieltges DW (2010). When parasites become prey: ecological and epidemiological significance of eating parasites. Trends Ecol Evol.

[CR34] Jørgensen L, Klemetsen A (1995). Food resource partitioning of Arctic charr, *Salvelinus alpinus* (L.) and three-spined stickleback, *Gasterosteus aculeatus* L., in the littoral zone of lake Takvatn in northern Norway. Ecol Freshw Fish.

[CR35] Kagami M, Van Donk E, de Bruin A, Rijkeboer M, Ibelings BW (2004). Daphnia can protect diatoms from fungal parasitism. Limnol Oceanogr.

[CR36] Kagami M, de Bruin A, Ibelings BW, Van Donk E (2007). Parasitic chytrids: their effects on phytoplankton communities and food-web dynamics. Hydrobiologia.

[CR37] Klemetsen A, Elliott JM (2010). Spatial distribution and diversity of macroinvertebrates on the stony shore of a subarctic lake. Int Rev Hydrobiol.

[CR38] Klemetsen A, Knudsen R (2013). Diversity and abundance of water birds in a subarctic lake during three decades. Fauna Norv.

[CR39] Klemetsen A, Muladal H, Amundsen P-A (1992). Diet and food consumption of young, profundal Arctic charr (*Salvelinus alpinus*) in Lake Takvatn. Nord J Freshw Res.

[CR40] Klemetsen A, Amundsen P-A, Dempson J, Jonsson B, Jonsson N, O'connell M, Mortensen E (2003). Atlantic salmon *Salmo salar* L., brown trout *Salmo trutta* L. and Arctic charr *Salvelinus alpinus* (L.): a review of aspects of their life histories. Ecol Freshw Fish.

[CR41] Klemetsen A, Aase BM, Amundsen P-A (2020). Diversity, abundance, and life histories of littoral chydorids (Cladocera: Chydoridae) in a subarctic European lake. J Crust Biol.

[CR42] Klemetsen A, Amundsen P-A, Grotnes PE, Knudsen R, Kristoffersen R, Svenning M-A (2002) Takvatn through 20 years: long-term effects of an experimental mass removal of Arctic charr, *Salvelinus alpinus*, from a subarctic lake. Ecology, behaviour and conservation of the charrs, genus *Salvelinus*. Springer, pp. 39–47

[CR43] Knudsen R, Gabler H, Kuris AM, Amundsen P-A (2001). Selective predation on parasitized prey—a comparison between two helminth species with different life-history strategies. J Parasitol.

[CR44] Kones JK, Soetaert K, van Oevelen D, Owino JO (2009). Are network indices robust indicators of food web functioning? A Monte Carlo approach. Ecol Modell.

[CR45] Koprivnikar J, Thieltges D, Johnson P (2023). Consumption of trematode parasite infectious stages: from conceptual synthesis to future research agenda. J Helminthol.

[CR46] Kuhn JA, Kristoffersen R, Knudsen R, Jakobsen J, Marcogliese DJ, Locke SA, Primicerio R, Amundsen P-A (2015). Parasite communities of two three-spined stickleback populations in subarctic Norway—effects of a small spatial-scale host introduction. Parasitol Res.

[CR47] Kuhn JA, Frainer A, Knudsen R, Kristoffersen R, Amundsen P-A (2016). Effects of fish species composition on *Diphyllobothrium* spp. infections in brown trout–is three-spined stickleback a key species?. J Fish Dis.

[CR48] Kuhn JA, Knudsen R, Kristoffersen R, Primicerio R, Amundsen P-A (2016). Temporal changes and between-host variation in the intestinal parasite community of Arctic charr in a subarctic lake. Hydrobiologia.

[CR49] Kuris AM (1990) Guild structure of larval trematodes in molluscan hosts: prevalence, dominance and significance of competition. In: Parasite Communities: Patterns and Processes. Springer, pp. 69–100

[CR50] Kuris AM, Lafferty KD (1994). Community structure: larval trematodes in snail hosts. Annu Rev Ecol Syst.

[CR51] Lafferty KD (2012). Biodiversity loss decreases parasite diversity: theory and patterns. Phil Trans R Soc B.

[CR52] Lafferty KD, Kuris AM (2009). Parasites reduce food web robustness because they are sensitive to secondary extinction as illustrated by an invasive estuarine snail. Phil Trans R Soc B.

[CR53] Lafferty KD, Kuris AM (2009). Parasitic castration: the evolution and ecology of body snatchers. Trends Parasitol.

[CR54] Lafferty KD, Shaw JC (2013). Comparing mechanisms of host manipulation across host and parasite taxa. J Exp Biol.

[CR55] Lafferty KD, Sammond D, Kuris AM (1994). Analysis of larval trematode communities. Ecology.

[CR56] Lafferty KD, Dobson AP, Kuris AM (2006). Parasites dominate food web links. Proc Natl Acad Sci USA.

[CR57] Lafferty KD, Hechinger RF, Shaw JC, Whitney K, Kuris AM, Collinge S, Ray C (2006). Food webs and parasites in a salt marsh ecosystem. Disease ecology: community structure and pathogen dynamics.

[CR58] Lafferty KD, Allesina S, Arim M, Briggs CJ, De Leo G, Dobson AP, Dunne JA, Johnson PT, Kuris AM, Marcogliese DJ (2008). Parasites in food webs: the ultimate missing links. Ecol Lett.

[CR59] Larsson P (1978). The life cycle dynamics and production of zooplankton in Øvre Heimdalsvatn. Ecography.

[CR60] Lillehammer A (1978). The plecoptera of Øvre Heimdalsvatn. Ecography.

[CR61] Lillehammer A (1978). The trichoptera of Øvre Heimdalsvatn. Ecography.

[CR62] Marcogliese DJ (2002). Food webs and the transmission of parasites to marine fish. Parasitology.

[CR63] Marcogliese DJ, Cone DK (1997). Food webs: a plea for parasites. Trends Ecol Evol.

[CR64] McKee KM, Koprivnikar J, Johnson PT, Arts MT (2020). Parasite infectious stages provide essential fatty acids and lipid-rich resources to freshwater consumers. Oecologia.

[CR65] McLaughlin JP (2018). The food web for the sand flats at Palmyra Atoll.

[CR66] Memmott J, Martinez N, Cohen J (2000). Predators, parasitoids and pathogens: species richness, trophic generality and body sizes in a natural food web. J Anim Ecol.

[CR67] Milardi M, Thomas SM, Kahilainen KK (2016). Reliance of brown trout on terrestrial prey varies with season but not fish density. Freshw Biol.

[CR68] Miura O, Kuris AM, Torchin ME, Hechinger RF, Chiba S (2006). Parasites alter host phenotype and may create a new ecological niche for snail hosts. Proc Royal Soc B.

[CR69] Monakov A (1972). Review of studies on feeding of aquatic invertebrates conducted at the Institute of Biology of Inland Waters, Academy of Science, USSR. J Fish Res Board Can.

[CR70] Morton DN, Lafferty KD (2022). Parasites in kelp-forest food webs increase food-chain length, complexity, and specialization, but reduce connectance. Ecol Monogr.

[CR71] Mouritsen KN, Poulin R, McLaughlin JP, Thieltges DW (2011). Food web including metazoan parasites for an intertidal ecosystem in New Zealand: ecological archives E092–173. Ecology.

[CR72] Nilsson A (1997) Aquatic insects of North Europe: A taxonomic handbook. Apollo books

[CR73] Nowosad P, Kuczyńska-Kippen N, Słodkowicz-Kowalska A, Majewska AC, Graczyk TK (2007). The use of rotifers in detecting protozoan parasite infections in recreational lakes. Aquat Ecol.

[CR74] Orlofske SA, Jadin RC, Johnson PT (2015). It’s a predator–eat–parasite world: how characteristics of predator, parasite and environment affect consumption. Oecologia.

[CR75] Poulin R, Thomas F (1999). Phenotypic variability induced by parasites: extent and evolutionary implications. Parasitol Today.

[CR76] Prati S, Henriksen EH, Knudsen R, Amundsen PA (2020). Seasonal dietary shifts enhance parasite transmission to lake salmonids during ice cover. Ecol Evol.

[CR77] Prati S, Henriksen EH, Knudsen R, Amundsen P-A (2020). Impacts of ontogenetic dietary shifts on the food-transmitted intestinal parasite communities of two lake salmonids. Int J Parasitol Parasites Wildl.

[CR78] Prati S, Henriksen EH, Smalås A, Knudsen R, Klemetsen A, Sánchez-Hernández J, Amundsen P-A (2021). The effect of inter-and intraspecific competition on individual and population niche widths: a four-decade study on two interacting salmonids. Oikos.

[CR79] Preston DL, Orlofske SA, McLaughlin JP, Johnson PT (2012). Food web including infectious agents for a California freshwater pond: ecological archives E093–153. Ecology.

[CR80] Preston DL, Jacobs AZ, Orlofske SA, Johnson PT (2014). Complex life cycles in a pond food web: effects of life stage structure and parasites on network properties, trophic positions and the fit of a probabilistic niche model. Oecologia.

[CR81] Rasconi S, Jobard M, Sime-Ngando T (2011). Parasitic fungi of phytoplankton: ecological roles and implications for microbial food webs. Aquat Microb Ecol.

[CR82] R Core Team (2020) R: a Language and environment for statistical computing. R Foundation for Statistical Computing, Vienna, Austria. https://www.R-project.org/

[CR83] Riede JO, Rall BC, Banasek-Richter C, Navarrete SA, Wieters EA, Emmerson MC, Jacob U, Brose U (2010). Scaling of food-web properties with diversity and complexity across ecosystems. Adv Ecol Res.

[CR84] Rovenolt FH, Tate AT (2022). The impact of coinfection dynamics on host competition and coexistence. Am Nat.

[CR85] Sánchez-Hernández J, Prati S, Henriksen EH, Smalås A, Knudsen R, Klemetsen A, Amundsen P-A (2022). Exploring temporal patterns in fish feeding ecology: are ontogenetic dietary shifts stable over time?. Rev Fish Biol Fish.

[CR86] Shaw JC, Henriksen EH, Knudsen R, Kuhn JA, Kuris AM, Lafferty KD, Siwertsson A, Soldánová M, Amundsen PA (2020). High parasite diversity in the amphipod *Gammarus lacustris* in a subarctic lake. Ecol Evol.

[CR87] Skoglund S, Knudsen R, Amundsen P-A (2013). Selective predation on zooplankton by pelagic Arctic charr, *Salvelinus alpinus*, in six subarctic lakes. J Icthyol.

[CR88] Soldánová M, Kuris AM, Scholz T, Lafferty KD (2012). The role of spatial and temporal heterogeneity and competition in structuring trematode communities in the great pond snail, *Lymnaea stagnalis* (L.). J Parasitol.

[CR89] Soldánová M, Georgieva S, Roháčová J, Knudsen R, Kuhn JA, Henriksen EH, Siwertsson A, Shaw JC, Kuris AM, Amundsen P-A, Scholz T, Lafferty KD, Kostadinova A (2017). Molecular analyses reveal high species diversity of trematodes in a sub-Arctic lake. Int J Parasitol.

[CR90] Sukhdeo MV (2012). Where are the parasites in food webs?. Parasit Vectors.

[CR91] Thieltges D, Jensen K, Poulin R (2008). The role of biotic factors in the transmission of free-living endohelminth stages. Parasitology.

[CR92] Thieltges DW, Reise K, Mouritsen KN, McLaughlin JP, Poulin R (2011). Food web including metazoan parasites for a tidal basin in Germany and Denmark: ecological archives E092–172. Ecology.

[CR93] Thieltges DW, Amundsen P-A, Hechinger RF, Johnson PT, Lafferty KD, Mouritsen KN, Preston DL, Reise K, Zander CD, Poulin R (2013). Parasites as prey in aquatic food webs: implications for predator infection and parasite transmission. Oikos.

[CR94] Thompson RM, Brose U, Dunne JA, Hall RO, Hladyz S, Kitching RL, Martinez ND, Rantala H, Romanuk TN, Stouffer DB (2012). Food webs: reconciling the structure and function of biodiversity. Trends Ecol Evol.

[CR95] Thorp JH, Covich AP (2009) Ecology and classification of North American freshwater invertebrates. Academic Press

[CR96] Vander Zanden MJ, Vadeboncoeur Y (2002). Fishes as integrators of benthic and pelagic food webs in lakes. Ecology.

[CR97] Vinagre C, Costa MJ, Wood SA, Williams RJ, Dunne JA (2019). Potential impacts of climate change and humans on the trophic network organization of estuarine food webs. Mar Ecol Prog Ser.

[CR98] Welsh JE, van der Meer J, Brussaard CP, Thieltges DW (2014). Inventory of organisms interfering with transmission of a marine trematode. J Mar Biol Assoc.

[CR99] Williams RJ, Martinez ND (2000). Simple rules yield complex food webs. Nature.

[CR100] Williams RJ, Martinez ND (2008). Success and its limits among structural models of complex food webs. J Anim Ecol.

[CR101] Williams RJ, Purves DW (2011). The probabilistic niche model reveals substantial variation in the niche structure of empirical food webs. Ecology.

[CR102] Wood SA, Russell R, Hanson D, Williams RJ, Dunne JA (2015). Effects of spatial scale of sampling on food web structure. Ecol Evol.

[CR103] Zander CD, Josten N, Detloff KC, Poulin R, McLaughlin JP, Thieltges DW (2011). Food web including metazoan parasites for a brackish shallow water ecosystem in Germany and Denmark: ecological Archives E092–174. Ecology.

