# Supplementary material for: Parasites alter food-web topology of a subarctic lake food web and its pelagic and benthic compartments
Source: Oecologia. 2024 Feb 7;204(2):257–77. doi: 10.1007/s00442-023-05503-w (PMC10907417; doi:10.1007/s00442-023-05503-w)
Supplement: Supplementary file 1 — Supplementary file1 (PDF 312 KB) [file 442_2023_5503_MOESM1_ESM.pdf]

Parasites alter food-web topology of a subarctic lake food web and its pelagic and benthic compartments: Electronic Supplemental Material

Shannon E. Moore[1], Anna Siwertsson[1], Kevin D. Lafferty[2], Armand M. Kuris[3], Miroslava Soldánová[4], Dana Morton[3], Raul Primicerio[1], Per-Arne Amundsen[1]

Corresponding author: Shannon E. Moore, moore.shannon.one@gmail.com

Authors' contributions: PAA, KDL, AMK, AS and SM conceptualized the study; AS, PAA, KDL, AMK and MS collected the data; SM analyzed the data and lead the writing of the manuscript, DM and RP supported in the conceptualization and data analyses; all the authors revised and commented on the manuscript; PAA provided funding and logistical support.

1. Department of Arctic and Marine Biology, Faculty of Biosciences, Fisheries and Economics, UiT The Arctic University of Norway, Tromsø, Norway
2. U.S. Geological Survey, Western Ecological Research Center, at Marine Science Institute, University of California, Santa Barbara, California, USA
3. Department of Ecology, Evolution, and Marine Biology, University of California, Santa Barbara, California, USA
4. Institute of Parasitology, Biology Centre, Czech Academy of Sciences, Branišovská 31, 370 05 České Budějovice, Czech Republic

Figure Legends

Fig. ESM.1 Food web of Takvatn's (a) free-living pelagic web, (b) free-living benthic web, and (c) free-living whole-lake web and their respective links. The nodes representing detritus and other non-living taxa are depicted in brown, the producers are depicted in green, and all other free-living taxa are depicted in blue. The links between these free-living nodes are blue

Fig. ESM.1

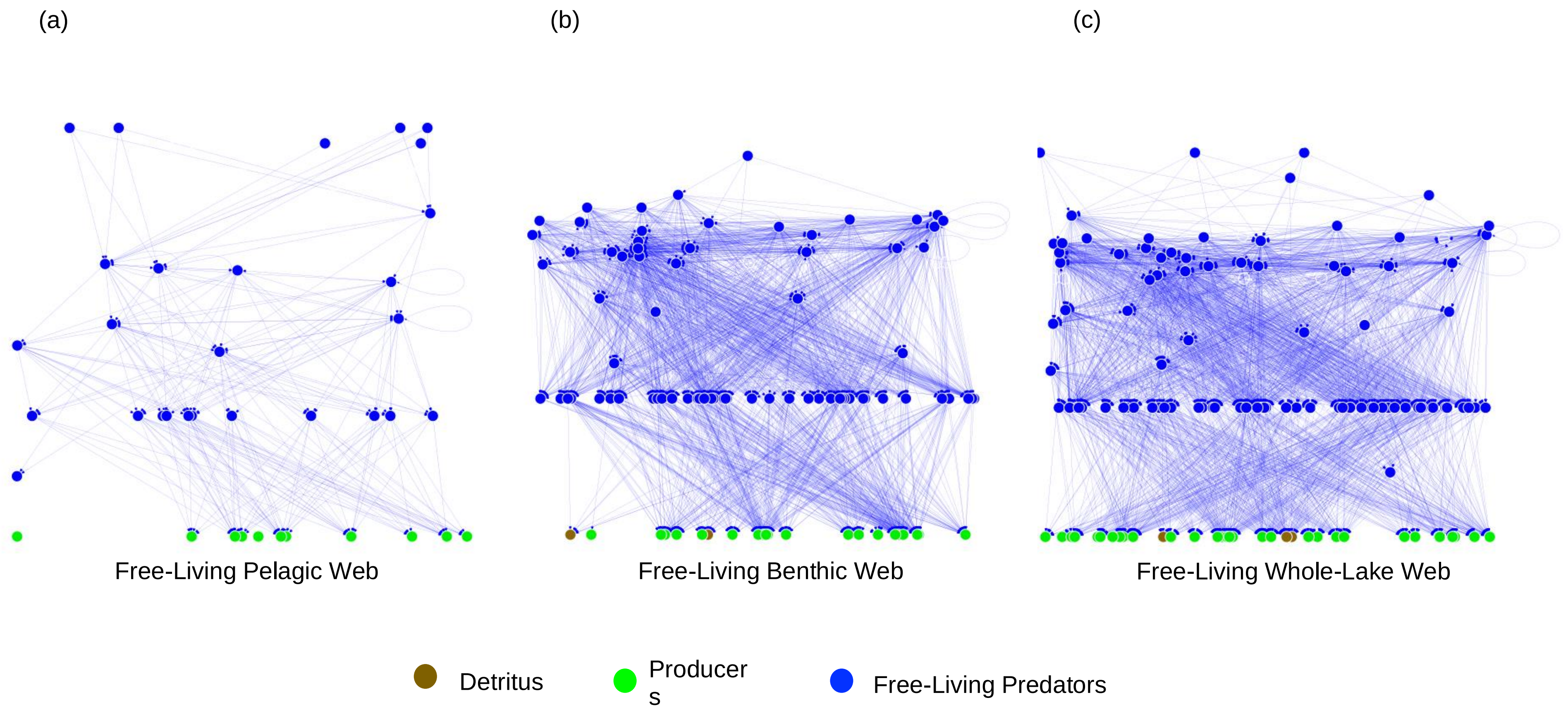
(a)
(b)
(c)
Free-Living Pelagic Web
Free-Living Benthic Web
Free-Living Whole-Lake Web
Detritus
Producer
s
Free-Living Predators
